# Supplementary material for: Impact of the COVID-19 nonpharmaceutical interventions on influenza and other respiratory viral infections in New Zealand
Source: Nat Commun. 2021 Feb 12;12:1001. doi: 10.1038/s41467-021-21157-9 (PMC7881137; doi:10.1038/s41467-021-21157-9)
Supplement: Supplementary file 3 — Reporting Summary [file 41467_2021_21157_MOESM3_ESM.pdf]

nature research

Corresponding author(s): Q. Sue Huang

Last updated by author(s): 2020-Dec-18

Reporting Summary

Nature Research wishes to improve the reproducibility of the work that we publish. This form provides structure for consistency and transparency in reporting. For further information on Nature Research policies, see our Editorial Policies and the Editorial Policy Checklist.

Statistics

For all statistical analyses, confirm that the following items are present in the figure legend, table legend, main text, or Methods section.

n/a	Confirmed	
☐	☒	The exact sample size (*n*) for each experimental group/condition, given as a discrete number and unit of measurement
☐	☒	A statement on whether measurements were taken from distinct samples or whether the same sample was measured repeatedly
☐	☒	The statistical test(s) used AND whether they are one- or two-sided<br>*Only common tests should be described solely by name; describe more complex techniques in the Methods section.*
☒	☐	A description of all covariates tested
☐	☒	A description of any assumptions or corrections, such as tests of normality and adjustment for multiple comparisons
☐	☒	A full description of the statistical parameters including central tendency (e.g. means) or other basic estimates (e.g. regression coefficient) AND variation (e.g. standard deviation) or associated estimates of uncertainty (e.g. confidence intervals)
☐	☒	For null hypothesis testing, the test statistic (e.g. *F*, *t*, *r*) with confidence intervals, effect sizes, degrees of freedom and *P* value noted<br>*Give P values as exact values whenever suitable.*
☒	☐	For Bayesian analysis, information on the choice of priors and Markov chain Monte Carlo settings
☒	☐	For hierarchical and complex designs, identification of the appropriate level for tests and full reporting of outcomes
☒	☐	Estimates of effect sizes (e.g. Cohen's *d*, Pearson's *r*), indicating how they were calculated

Our web collection on statistics for biologists contains articles on many of the points above.

Software and code

Policy information about availability of computer code

Data collection	REDCap 10.0.19
Data analysis	Analyses were performed in Stata 16.1 (StataCorp LLC).

For manuscripts utilizing custom algorithms or software that are central to the research but not yet described in published literature, software must be made available to editors and reviewers. We strongly encourage code deposition in a community repository (e.g. GitHub). See the Nature Research guidelines for submitting code & software for further information.

Data

Policy information about availability of data

All manuscripts must include a data availability statement. This statement should provide the following information, where applicable:

- Accession codes, unique identifiers, or web links for publicly available datasets
- A list of figures that have associated raw data
- A description of any restrictions on data availability

Anonymized raw data and Stata syntax are used to produce all the analyses, figures and tables. Source data are provided with this paper. All requests for raw and analyzed data will be reviewed by the corresponding authors to verify whether the request is subject to any intellectual property or funder or confidentiality obligations.

Field-specific reporting

Please select the one below that is the best fit for your research. If you are not sure, read the appropriate sections before making your selection.

- [x] Life sciences
- [ ] Behavioural & social sciences
- [ ] Ecological, evolutionary & environmental sciences

For a reference copy of the document with all sections, see nature.com/documents/nr-reporting-summary-flat.pdf

Life sciences study design

All studies must disclose on these points even when the disclosure is negative.

Sample size	We did not perform statistical analysis to predetermine sample sizes. We used multiple surveillance systems established previously to monitor annual influenza activity:<br>• Severe Acute Respiratory Illness (SARI) surveillance, ICD-coded influenza hospitalizations: assessing severe influenza requiring hospitalization, ICU admissions and in-hospital deaths<br>• Sentinel general practice based influenza-like illness surveillance: assessing moderate influenza requiring GP consultations.<br>• SHIVERS-II&III (the second and third iterations of the Southern Hemisphere Influenza and Vaccine Effectiveness Research and Surveillance program): assessing mild influenza not requiring medical attentions at a community level<br>• Laboratory-based influenza surveillance: laboratory confirmation of influenza for those samples ordered either by clinicians or surveillance staff<br><br>A combination of these datasets provides a comprehensive understanding of the impact of influenza at varying levels of disease severity in 2020. Additionally, when the 2020 data compares with the data from previous five years, it allows differentiation of influenza circulation pattern in 2020 vs previous years. New Zealand's influenza surveillance systems represent one of the most comprehensive systems in the world and we used almost all influenza related datasets collected in New Zealand in this analysis. These datasets are sufficient for this analysis.
Data exclusions	All patients and samples were included. No data exclusions were performed
Replication	We used multiple surveillance systems to monitor influenza activity in 2020. All of these systems replicated the same results that there was no annual laboratory-confirmed influenza outbreak or epidemic detected during the 2020 winter season. Computer codes are available to reproduce all the analyses, figures and tables.
Randomization	Our study is an observation study, so no randomization is needed.
Blinding	Laboratory testing on respiratory samples were performed independently by laboratory staff who were not involved in sample collection and clinical meta-data collection. The data analysis were performed by data analysts who were not involved in sample collection and testing.

Reporting for specific materials, systems and methods

We require information from authors about some types of materials, experimental systems and methods used in many studies. Here, indicate whether each material, system or method listed is relevant to your study. If you are not sure if a list item applies to your research, read the appropriate section before selecting a response.

Materials & experimental systems

n/a	Involved in the study	
x		Antibodies
x		Eukaryotic cell lines
x		Palaeontology and archaeology
x		Animals and other organisms
	x	Human research participants
x		Clinical data
x		Dual use research of concern

Methods

n/a	Involved in the study	
x		ChIP-seq
x		Flow cytometry
x		MRI-based neuroimaging

Human research participants

Policy information about studies involving human research participants

Population characteristics	SARI surveillance: SARI study sites are located within two District Health Boards of the Auckland region of NZ: ADHB and CMDHB. This is a predominantly urban population of ~1Million people, with a wide spectrum of socio-economic, cultural, ethnic and demographic groups broadly similar to the New Zealand population.<br><br>ILI surveillance: The population-based surveillance for influenza-like illness (ILI) among persons enrolled in sentinel general practices (~90) who seek medical consultations has been in operation since 1990, usually covering ~10% of the NZ population with a wide spectrum of socio-economic, cultural, ethnic and demographic groups broadly similar to the New Zealand population.<br><br>SHIVERS-II: Individuals aged 20-69 years were randomly selected from those healthy individuals listed in the GP's primary management system<br>SHIVERS-III (WellKiwis): Eligible participants will include newborns and their mothers of all NZ prioritised ethnicity (Māori, Pacific, Asian, or European/other), residing in the Wellington region. For inclusion in the study, a participant must:<br>(1) live in the greater Wellington Region (study location),<br>(2) expect to remain in the study area for the next 2 years,<br>(3) infants born at ≥32 weeks' gestation and aged ≤2 months at enrolment,<br>(4) have a legal guardian to complete informed consent procedures and to respond to survey and facilitate sample collection.
Recruitment	SARI surveillance: NZ has an excellent public health infrastructure. All residents of ADHB and CMDHB with acute respiratory illnesses are channeled to ADHB and CMDHB hospitals. Thus, it provides robust population-based influenza incidence rate<br><br>ILI surveillance: The participation for sentinel general practice is voluntary. It may result self-selection bias. In 2020, sentinel general practice-based ILI surveillance was not operated in a usual way due to the COVID-19 response. Instead of visiting sentinel GPs for medical consultations, many ILI patients would visit one of the community-based testing centres. Additionally, national sentinel GP-based ILI surveillance requires swabbing from an ILI patient. This may contribute to less GP participation (18-57% of the usual participation rate over the winter period in 2020) in the COVID-19 pandemic situation. These factors would contribute to lower consultation, reporting and detection of influenza and other respiratory viruses compared with previous years.<br><br>SHIVERS-II cohort: Only ~10% of individuals aged 20-69 years were randomly selected from those healthy individuals listed in the GP's primary management system participated in the study. It has potentially self-selection bias (such as more educated european participants)<br><br>SHIVERS-III (WellKiwis) cohort: Potential participants were referred by midwives or self-referral or direct recruitment by study staff. It may have potentially self-selection bias (at the moment the number of participants is still low).<br><br>Laboratory-based surveillance: This laboratory network tests specimens ordered by clinicians for hospital inpatients and outpatients during normal clinical practice (serving ~70% of the NZ population). Sample collection is based on clinician's judgement, rather than systematic sampling approach. This may result in selection bias. In 2020 during the COVID-19 laboratory response, some labs may have prioritised testing for SARS-CoV-2 over influenza and other respiratory viruses. This would contribute to lower testing and reporting compared with previous years.
Ethics oversight	Ethical approval was obtained for the SHIVERS (including SARI and ILI surveillance), SHIVERS-II and III cohort studies from the NZ Northern A Health and Disability Ethics Committee (NTX/11/11/102). The ICD-coded influenza hospitalisation data and laboratory-based respiratory virus surveillance data are part of public health surveillance in NZ. It is conducted in accordance with the Public Health Act and thus ethics committee approval was not needed for collection or use of these data.

Note that full information on the approval of the study protocol must also be provided in the manuscript.
